# Direct detection of lipid A on intact Gram-negative bacteria by MALDI-TOF mass spectrometry

**DOI:** 10.1016/j.mimet.2015.12.004

**Published:** 2016-01

**Authors:** Gerald Larrouy-Maumus, Abigail Clements, Alain Filloux, Ronan R. McCarthy, Serge Mostowy

**Affiliations:** a MRC Centre for Molecular Bacteriology and Infection, Department of Life Sciences, Faculty of Natural Sciences, Imperial College London, London, UK; b MRC Centre for Molecular Bacteriology and Infection, Department of Medicine, Imperial College London, London, UK

**Keywords:** Endotoxins, Mass spectrometry, Gram-negative bacteria

## Abstract

The purification and characterization of Gram-negative bacterial lipid A is tedious and time-consuming. Herein we report a rapid and sensitive method to identify lipid A directly on intact bacteria without any chemical treatment or purification, using an atypical solvent system to solubilize the matrix combined with MALDI-TOF mass spectrometry.

## Highlights

• Gram-negative bacterial lipid A can be directly detected on intact bacteria by MALDI-TOF MS.
• No extractions or purification steps are required.
• The method is rapid (< 5 min), and only requires about 10^4^–10^5^ bacteria.

Lipopolysaccharide (LPS), composed of the O-antigen, the oligosaccharide core and the lipid A, is the major surface glycolipid located in the outer membrane of Gram-negative bacteria (Raetz and Whitfield, 2002, Caroff and Karibian, 2003). Although significant efforts have been made to isolate and purify lipid A, the whole extraction process is time-consuming, involves the use of highly hazardous reagents, and requires a large volume of bacteria cultures (Westphal and Luderitz, 1953, Westphal et al., 1981, El Hamidi et al., 2005, Sturiale et al., 2005, De Castro et al., 2010). In addition, the requirement for chemical hydrolysis of LPS into its lipid A and polysaccharide components results in the loss or potential modification of the lipid A moiety, such as dephosphorylation or *O*-deacylation, as well as loss of labile residues. Maintenance of these modifications is crucial to determine the native structure of the lipid A and its consequence in the host immune response (Shimazu et al., 1999, Kawahara et al., 2002, Pier, 2007, DeMarco and Woods, 2011). For this reason, we decided to investigate an alternative methodology to allow detection of lipid A directly on intact Gram-negative bacteria without any chemical treatment or purification steps. This method allows users to obtain data on the main lipid A forms in a sample.

The Gram-negative, medically relevant strains used in this study are: *Shigella sonnei* 53G, *Shigella flexneri* M90T, *Klebsiella pneumoniae* B5055, *Pseudomonas aeruginosa* PAK, enterohemorrhagic *Escherichia coli* EDL933 and enteroinvasive *E. coli* 1457-75. Strains were cultivated under aerobic conditions in Luria Broth medium at 37 °C overnight at 180 rpm. Bacteria were handled within a Class-II safety-level cabinet equipped with UV light source and HEPA filters and placed into a 1.5 ml microtube by 1 ml of culture medium prior to heat-inactivation for 1 h at 90 °C in a water-bath. The heat-inactivated bacteria were then washed three times with 0.5 ml of double distilled water at 9000 ×* g* for 5 min and suspended in double distilled water at a final concentration of about 10^4^ to 10^5^ bacteria per μl. This was estimated using a “Thoma cell” counting method (Strober, 2001). Prior to mass spectrometry analysis, the 2, 5-dihydroxybenzoic acid (DHB) matrix was used at a final concentration of 10 mg/ml in chloroform/methanol (CHCl_3_/MeOH) in a ratio 90:10 *v*/*v*. 0.5 μl of bacteria solution and 0.5 μl of the matrix solution were deposited on the target, mixed with a micropipette and dried gently under a stream of air. After optimization, this solvent system and ratio were selected in order to selectively ionize lipid A. MALDI-TOF MS analysis was performed on a 4800 Proteomics Analyzer (with TOF–TOF Optics, Applied Biosystems) using the reflectron mode. Samples were analyzed operating at 20 kV in the negative ion mode using an extraction delay time set at 20 ns. Typically, spectra from 500 to 2000 laser shots were summed to obtain the final spectrum. All experiments were carried out in three independent bacterial cultures and in three technical replicates. The negative control consists of 0.5 μl of double distilled water and 0.5 μl of the matrix solution. Mass spectrometry data were analyzed using Data Explorer version 4.9 from Applied Biosystems. For all bacterial species, the negative mass spectrum was scanned between *m*/*z* 1000 and 2200.

**Fig. 1:**
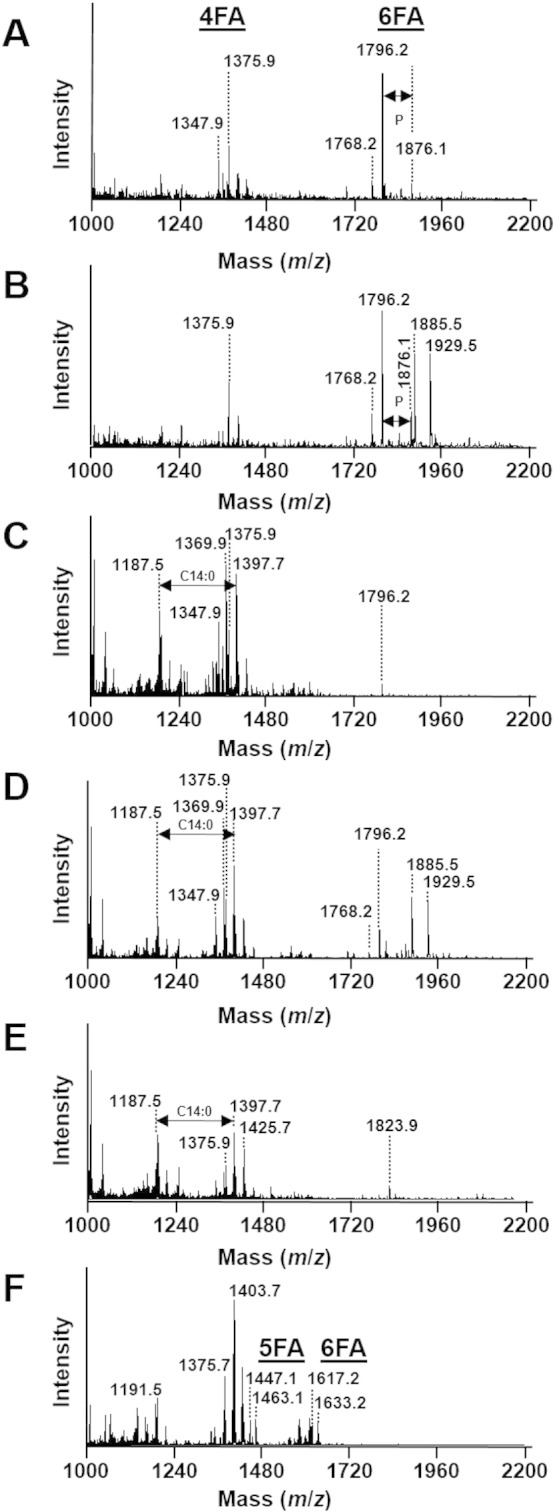
Negative ion mode MALDI-TOF MS spectra of intact heat-inactivated *S. sonnei* (A), *S. flexneri* (B), enterohemorrhagic *E. coli* (C), enteroinvasive *E. coli* (D), *K. pneumonia* (E), *P. aeruginosa* (F). (FA: fatty acid).

**Fig. 2:**
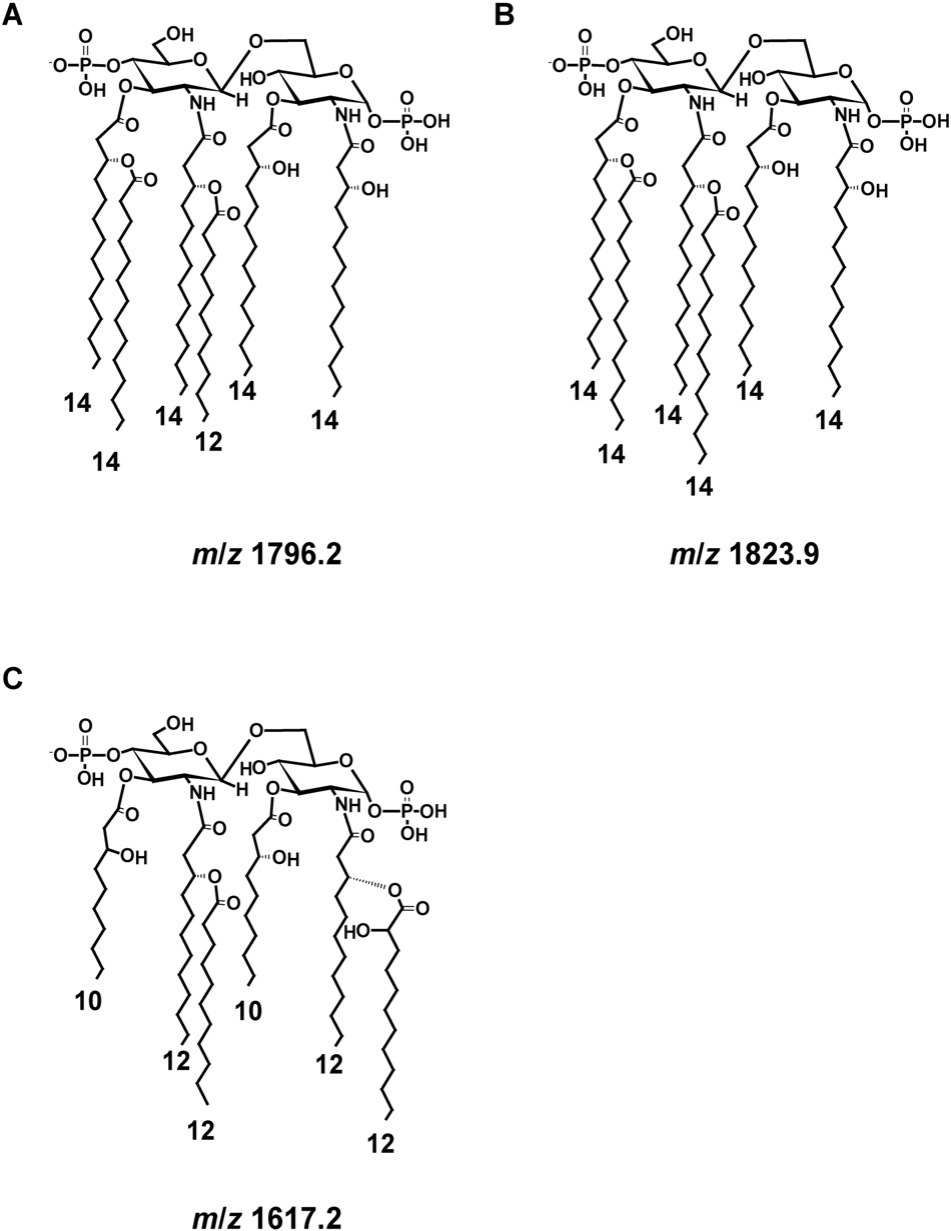
Structure of the lipid A analyzed in the study. (A) S*higella* species and *E. coli*, (B) *K. pneumoniae*, (C) *P. aeruginosa*.

In *S. sonnei*, the mass spectrum is dominated by two sets of peaks assigned to tetra-acyl lipid A and hexa-acyl lipid A (Fig. 1A). The peak at *m*/*z* 1796.2 corresponds to hexa-acyl diphosphoryl lipid A containing four C14:0 3-OH, one C14:0 and one C12:0 (Paciello et al., 2013) (Fig. 2A). The peak at *m*/*z* 1768.2, distant of 28 mass units, characterizes also hexa-acyl diphosphoryl lipid A but with globally two methylene units less compared to the peak at *m*/*z* 1796.2. We suggest that this species represents a hexa-acyl diphosphoryl lipid A with one C14:0 acyl chain substituted with a C12:0 acyl chain. The peak at *m*/*z* 1876.1 is assigned to hexa-acyl triphosphoryl lipid A due to the increase by 80 mass units to the peak at *m*/*z* 1796.2, which corresponds to one phosphate group. The peaks at *m*/*z* 1347.9 and *m*/*z* 1375.9 are tentatively assigned to tetra-acyl diphosphoryl lipid A (Sturiale et al., 2011). However, further investigations are required to determine the exact structure of these identified molecules. In *S. flexneri* (Fig. 1B), all of these peaks are also observed and assigned to tetra-acyl and hexa-acyl diphosphoryl lipid A. For both species of *Shigella*, the masses observed are in accordance to the reference mass spectra of purified lipid A from these species (De Castro et al., 2010, Sturiale et al., 2011). Interestingly, this method also revealed two peaks at *m*/*z* 1885.5 and *m*/*z* 1929.5 present in *S. flexneri* which are absent in *S. sonnei*. These two molecular species do not correspond to extra lipid A modifications commonly found such as ethanolamine phosphate, aminoarabinose, *N*-acetylglucosamine, or extra-acylation. Further investigations are required to confidently assign these peaks. Detection of these extra-peaks could potentially be used as a tool to discriminate these two closely related species for diagnostic purposes.

For EHEC (Fig. 1C), the mass spectrum is dominated by three sets of peaks. The peak at *m*/*z* 1796.2 corresponds to hexa-acyl diphosphoryl lipid A containing four C14:0 3-OH, one C14:0 and one C12:0 (Fig. 2A). As discussed for lipid A from *Shigella* species, the ions centered at *m*/*z* 1375.9 are tentatively assigned to tetra-acyl diphosphoryl lipid A (Sturiale et al., 2011). However, further investigations are required to determine the structure of these molecules. In addition, three peaks are present at *m*/*z* 1187.5; *m*/*z* 1369.9 and *m*/*z* 1397.7, which are not directly related to any other known modifications of the lipid A but predicted to belong to lipid molecules as the differences between them (182 and 210 mass units from *m*/*z* 1187.5 to *m*/*z* 1369.9 or *m*/*z* 1397.7) can be assigned to one C12:0 and C14:0, respectively. For EIEC (Fig. 1D), the mass spectrum is dominated by four sets of peaks. As for *Shigella* species and EHEC, the same peaks are observed in EIEC including hexa-acyl lipid A at *m*/*z* 1768.2 and *m*/*z* 1796.2 which is similar to the ones observed for purified lipid A (El Hamidi et al., 2005, Zhou et al., 2010). In addition, as observed for *S. flexneri*, for EIEC this method reveals two sets of unknown molecular species at *m*/*z* 1885.5 and *m*/*z* 1929.5.

In the case of *K. pneumoniae*, the negative mass spectrum is dominated by three sets of peaks centered at *m*/*z* 1187.5, *m*/*z* 1397.7 and *m*/z 1823.9. The peak *m*/*z* 1823.9 is assigned to hexa-acyl diphosphoryl lipid A containing four C14:0 3-OH and two C14:0 (Clements et al., 2007) (Fig. 2B). This methodology also reveals three peaks at *m*/*z* 1187.5; *m*/*z* 1397.7 and *m*/*z* 1425.7. These molecular ions are not directly related to any of the other known modifications of the lipid A as described earlier but predicted to belong to lipid molecules as the differences between them (210 and 238 mass units from *m*/*z* 1187.5 to *m*/*z* 1397.7 or *m*/*z* 1425.7) can be assigned to one C14:0 and one C16:0, respectively.

In the case of *P. aeruginosa*, the negative mass spectrum is dominated by three sets of peaks centered at *m*/*z* 1191.5, *m*/*z* 1403.7 and *m*/*z* 1617.2. The peaks at *m*/*z* 1447.1 and *m*/*z* 1463.1 are assigned to penta-acyl diphosphorylated lipid A which corresponds to the presence of one C10:0 3-OH, three C12:0 2- or 3-OH, and one C12:0 (Ernst et al., 2006, Moskowitz et al., 2012). The peaks at *m*/*z* 1617.2 and *m*/*z* 1633.2 are assigned to hexa-acyl diphosphorylated lipid A, due to the addition of 170 mass units which corresponds to one C10:0 3-OH fatty acid (Fig. 2C). This new methodology reveals additional peaks at *m*/*z* 1191.5, and two sets of molecular ions with differences of 28 and 56 mass units corresponding to two and four methylene units respectively (*m*/*z* 1375.7 to *m*/*z* 1403.7, and from *m*/*z* 1551.6 to *m*/*z* 1607.6) typifying the lipid nature of these molecules, which have not been assigned yet.

This new and sensitive application applied here allows one to characterize lipid A and its modifications directly by MALDI-MS analysis of intact heat-inactivated bacteria without any extraction or purification steps. Moreover, our analysis requires only about 10^4^ to 10^5^ bacteria. This represents a major advancement for the analysis of lipid A from Gram-negative bacteria allowing the study of native lipid A and its modifications directly on bacteria without large starting volumes or the use of chemical reagents or harsh treatments. This method may therefore reveal previously uncharacterized modifications as well as allowing rapid analysis of native lipid A under different conditions.

This work was supported by the Department of Life Sciences from the Faculty of Natural Sciences Imperial College London, UK. Work in the SM laboratory is supported by a Wellcome Trust Research Career Development Fellowship (WT097411MA) and the Lister Institute of Preventive Medicine. RRMC is supported by BBSRC grant (BB/L007959/1). We thank Ms. Sina Krokowski (MRC-CMBI) for the preparation of bacterial cultures. We thank Dr. Frankie Bolt, Imperial College London, and Dr. Germain Puzo (CNRS, IPBS-Toulouse) for their helpful discussion and careful reading of the manuscript. The authors declare no competing financial interest.
